# Hepatitis B Response of Premature Infants after Primary and Booster Immunisation with a Diphtheria-Tetanus-Acellular Pertussis-Hepatitis B-Inactivated Poliovirus/*Haemophilus Influenzae* Type B Vaccine

**DOI:** 10.1155/2010/802503

**Published:** 2010-04-12

**Authors:** Felix Omeñaca, Jose Garcia-Sicilia, Reyes Boceta, Pilar García-Corbeira

**Affiliations:** ^1^Neonatal Unit, Department of Neonatology, La Paz Hospital, Castellana 261, 28046 Madrid, Spain; ^2^Department of Paediatrics, La Paz Hospital, 28046 Madrid, Spain; ^3^Medical Department, GlaxoSmithKline, Tres Cantos, Madrid, Spain

## Abstract

A range of schedules are recommended for hepatitis B vaccination of premature infants. This open-label study (217744/083) compared the immune response of premature (*N* = 94) and full-term infants (*N* = 92) to hepatitis B antigen following primary administration of hexavalent DTPa-HBV-IPV/Hib vaccine at 2–4–6 months and a booster dose at 18 months. Anti-HBsAg antibodies were determined before and one month after primary and booster doses. There were no significant differences in postprimary seroprotection rates (anti-HBsAg >10 mIU/mL; preterm 93.4%; full-term 95.2%) or geometric mean concentrations (634 versus 867 mIU/ml), and neither appeared to be related to gestational length or birth weight. Prebooster seroprotection rates were 75 and 80.6%, respectively. Six premature infants did not respond to primary and booster doses. Primary and booster vaccinations with DTPa-HBV-IPV/Hib elicit satisfactory anti-HBsAg responses in preterm infants, which are not influenced by gestational age or birth weight. This schedule and vaccine will greatly facilitate the immunisation of premature infants.

## 1. Introduction

Infection with hepatitis B virus (HBV) persists as a worldwide public health problem, with vertical transmission of HBV being responsible for approximately one third of all new cases of hepatitis B. Childhood hepatitis B immunisation has significantly reduced the incidence and prevalence of HBV infection [1], and currently more than 160 countries use hepatitis B vaccine in their national immunisation programmes. Exciting new global initiatives have been implemented that allow the poorest countries in the world to afford this vaccine [2]. 

The number of infants born prematurely has risen in the last 15 years, and recent advances in the care of premature infants have substantially increased their survival rates [3]. It is now thought that prematurity, rather than a specific gestational age or birth weight, is more predictive of immunologic HBV response compared with full-term infants. The latest recommendation is that medically stable preterm or low-birth weight babies (weighing >2000 g) who are born to hepatitis B surface antigen (HBsAg) negative mothers should receive HBV at birth or shortly thereafter [4]. 

Concern among parents and paediatricians about the number of injections required during each immunisation visit has contributed to the observation that routine paediatric vaccination is often delayed in preterm infants [5, 6]. However, several combined vaccines are now available which reduce the required number of injections and medical visits. The acceptability of multiple immunisations and increasing the coverage of each vaccine can thereby be achieved.

We have previously shown preterm infants less than 37 weeks of gestational age to display satisfactory immune response to all component antigens of a hexavalent diphtheria-tetanus-acellular pertussis-hepatitis B-inactivated poliovirus-*Haemophilus influenzae* type B vaccine (DTPa-HBV-IPV/Hib), with seroprotection/vaccine response rates generally similar to those seen in full-term infants following primary vaccination and a booster dose [7–9]. This paper details the results of a further analysis of the immune response in the preterm cohort to the hepatitis B component of this hexavalent vaccine, including a follow-up at 18 months and a specific focus on the nonresponders.

## 2. Materials and Methods

### 2.1. Study Design

This was an open-label study with two parallel groups: preterm subjects (<37 weeks' gestation) and a control group of full-term infants. The study protocol was approved by the Clinical Investigation Research Board of La Paz Hospital and conducted in accordance with Good Clinical Practice Guidelines. Written informed consent was obtained from the parents or guardians of all subjects prior to enrolment. Trial number: 217744/083 (DTPa-HBV-IPV-083).

### 2.2. Subjects

Preterm subjects were stratified according to clinical characteristics including length of gestation, birth weight, pre- and postnatal steroid use, need for red blood cell transfusion, and weight at 6 months. Length of gestation was determined by date of the last menstrual period and/or early ultrasound scan and subsequently confirmed by neonatal examination. Exclusion criteria comprised major congenital defects or serious chronic illnesses, severe neurologic damage or nontreatable convulsions; known or suspected immune dysfunction, HIV positive or hepatitis B surface antigen (HBsAg) positive mother, acute disease or rectal temperature ≥38°C (immunization deferred), a history of allergic reaction to any of the vaccine components, apnea episode within 7 days of vaccination, steroid therapy 30 days before the first vaccine dose, any immunoglobulin therapy within 2 months before enrollment or during the trial, administration of any vaccine or experimental drug or vaccine during the 30 days after/before the administration of the study vaccine.

### 2.3. Vaccines

All subjects were primed with a DTPa-HBV-IPV/Hib vaccine [*Infanrix hexa, *GlaxoSmithKline (GSK) Biologicals, Rixensart, Belgium] at 2, 4, and 6 months and received a booster dose with the same vaccine at 18 months of age. The vaccine was administered as a 0.5 mL intramuscular injection into the right anterolateral thigh. 

Each dose of DTPa-HBV-IPV/Hib vaccine contained ≥30 international units (IU) of diphtheria toxoid, ≥40 IU of tetanus toxoid, 25 *μ*g of adsorbed pertussis toxin, 25 *μ*g of adsorbed filamentous hemagglutinin, 8 *μ*g of adsorbed pertactin, 10 *μ*g of HBsAg, 40, 8, and 32 D-antigen units of polio virus types 1, 2, and 3, respectively, and 0.7 mg of aluminum as salts. The lyophilized Hib vaccine pellet contained 10 *μ*g of Haemophilus influenzae type b polysaccharide conjugated to 20 to 40 *μ*g of tetanus toxoid, 0.12 mg of aluminium phosphate, and 10 mg of lactose. 

For infants not responding to primary and booster doses (anti-HBs <10 mIU/mL), three further 10 *μ*g doses of hepatitis B recombinant vaccine (*Engerix-B*; GSK Biologicals) could be administered in the third year of life. If the anti-HBs titre was above 100 mIU/mL after the second additional dose (6th in total), then the third dose was not given. If it was below 100 mIU/mL, then the third dose was administered.

### 2.4. Immunogenicity Analysis

Blood samples were drawn before the first dose and one month after the third in primary course and before and one month after the booster dose. Serum samples were stored at −20°C until analysis at GSK Biologicals. Antibodies against HBV were determined using ELISA (enzyme-linked immunosorbent assay), with an assay cut-off of 10 mIU/mL for HBsAg.

### 2.5. Statistical Analysis

Statistical analysis of the immunogenicity results was performed on the according-to-protocol (ATP) cohort. Seroprotection rates and antibody geometric mean concentrations (GMCs) were calculated with exact 95% confidence intervals (CI) at each time point. Differences in seroprotection rates between the preterm and full-term groups were compared using Fisher's exact test.

## 3. Results

A total of 186 infants were enrolled (94 preterm and 92 full-term), of whom 93 and 89 infants, respectively complied with the criteria for inclusion in the ATP cohort for analysis of immunogenicity of the primary vaccination course. The demographic and neonatal characteristics of the infants have been previously described [7]. The mean age at the time of first immunisation was 8.6 ± 0.63 weeks (range 8–11 weeks) and 8.2 ± 0.8 (range 6–11 weeks), respectively. 

Of the 155 infants who received booster doses, 152 subjects were included in the ATP cohort for analysis of immunogenicity of the booster dose (84 prematurely born subjects and 68 full-term subjects). Mean age at the time of booster vaccination was 18.2 ± 0.47 months (range 18–20 months) and 18.3 ± 0.55 months (range 18–20 months), respectively.

### 3.1. Postprimary Immunogenicity Results

The mean immunogenicity data after primary course and before and after booster dose are included in Table 1. A total of 93.4% of the preterm and 95.2% of the full-term infants responded to primary vaccination (anti-HBs ≥10 mIU/mL). Although the GMCs after the primary course were numerically lower in the preterm group, the differences were not significant.

The immunogenicity results by gestational age are shown in Table 2. All subjects with a gestation below 31 weeks were seroprotected compared with 83.3% and 92.6% in the groups with a higher gestational age. However, with respect to GMCs, a numerically higher response was seen in the groups aged 34–36 weeks and 28–30 weeks but the differences were not significantly different. After the booster dose, the seroprotection rates were lower for the groups with higher gestational age but GMCs retained a similar pattern to that observed after the primary course.

A nonconsistent response was also seen in the analysis according to birth weight (Table 3), where 92% of subjects with the lowest birth weight were protected compared with all babies with birth weight 1000–1500g. Only 82.4% of those weighing more than 2000 g at birth were seroprotected. No specific pattern of GMC response was observed. Similarly, there was no significant difference according to babies with (*N* = 15) or without (*N* = 76) intrauterine growth restriction (seroprotection rates: 86.6 and 94.7%, resp.), or with (*N* = 67) or without (*N* = 24) prenatal steroids (seroprotection rates: 91 and 100%, resp.).

Post-primary anti-HBs seroprotection rates and GMCs were numerically lower in the nine subjects who had received postnatal steroids (88.9% and 188.1, resp.) than the 81 subjects who had not received them (93.9% and 724.6, resp.), but the differences were not significant.

Blood transfusion during the neonatal period did not significantly affect seroprotection rates (anti-HBs ≥10 mIU/mL) or GMCs [transfused infants: 94.7% (95% CI 82.3–99.4) and 486.6 (95% CI 280.0–845.5) mIU/ml, respectively; no transfusion: 92% (95% CI 81.8–97.9) and 766.8 (95% CI 542.8–1298.7) mIU/mL, resp.) in all babies.

Considering weight at 2 and 6 months as a variable independent of gestational age and birth weight, no significant patterns of differences were observed in seroprotection rates (Table 4).

### 3.2. Prebooster Persistence and Booster Response

Before the booster dose, both the seroprotection rates of anti-HBs GMCs were low in both the pre- and full-term groups (Table 1). Seroprotection rose to 91.6 and 98.5%, respectively one month after the booster dose and the GMCs increased to 1771 and 1965 mIU/mL, respectively. There were no significant differences between the responses in the preterm and full-term groups.

### 3.3. Nonresponders

Six preterm infants (6.59%) did not respond to primary immunisation and also failed to respond to the booster dose (anti-HBs ≥10 mIU/mL; Table 5). All of these infants had a gestational age above 31 weeks. Two babies weighed less than 1000 g at birth and had severe intrauterine growth restriction (IUGR; 32 weeks/600 g and 31 weeks/800 g), but the other four babies did not have any risk factors. 

One infant responded to the 5th HBV dose, two to the 6th, and one to the 7th. Two babies (2.19%) never responded. One was an infant with IUGR who had anti-HBs antibodies before vaccination and the other although born prematurely at 33 weeks weighed 2000 g and did not have risk factors. These 6 infants did show a good response to the other antigens included in the DTPa-HBV-IPV/Hib vaccine.

## 4. Discussion

The American Academy of Pediatrics (AAP) recommends that all infants receive hepatitis B vaccine at birth or before discharge from the hospital. In infants weighing less than 2000 g and born to HbsAg negative mothers, the first dose of hepatitis B vaccine is recommended at 30 days [4]. 

Immunogenicity studies indicate that premature children generally respond less well to hepatitis B vaccination than full-term infants, both in terms of seroprotection rates [10–15] and GMCs [14, 16–21]. Our results are consistent with these findings, although the seroprotection rate in preterm subjects was not significantly different to that in full-term subjects.

Analysis according to birth weight in premature infants has suggested a relationship between reduced immunological response and low birth weight [16, 20, 22, 23]. Interestingly, in our study, babies under 1000 g achieved higher seroprotection rates than those children with a birth weight in excess of 2000 g, but their GMC response was lower. 

Gestational age seems to be a much more objective parameter than weight for assessing immune response in premature babies. After birth dosing, both seroprotection rates and GMCs are generally lower in babies with a reduced gestational age [16, 20, 21, 23] but this trend reverses as the chronological age at the time of starting vaccination increases [24]. In our study, all children with a gestational age below 31 weeks responded to immunisation in the two groups with a greater gestational age. The response in terms of GMCs was however irregular. 

Our findings strengthen the notion that the immunological response in premature babies depends on postnatal age of vaccination and not on birth weight or gestational age. Indeed, studies in term infants have demonstrated that very young infants (up to 2 months) achieve lower GMCs after three doses of hepatitis B vaccine compared to infants who are at least 3 months of age at the time of first vaccination [25].

Low-birth weight has been reported to be associated with an inadequate immune response to early hepatitis B vaccination in premature infants by some workers [16], but others have either shown no effect [19] or the opposite [26]. Similar anomalies have been seen when considering gestational age [19, 26] and the postnatal use of corticosteroids [16, 26, 27]. In our study all of the nonresponders had a gestational age of at least 31 weeks and, except for two babies with severe IUGR, were of appropriate weight. Nine infants received postnatal corticosteroids and although they responded to a lesser extent to hepatitis B vaccination, the differences were not significant. Only one of these babies was a non-responder. Two children in our cohort received transfusions (1 or 6) in the neonatal period, coinciding with severe intrauterine restriction, but no differences in response were identified between the transfused and nontransfused infants. 

Other factors linked to nonresponse to hepatitis B vaccination in premature babies include hyperbilirubinaemia [28], sepsis [16, 19, 26], and the presence of specific antibodies transferred by the mother through the placenta. All of the children in our study who did not respond to HBsAg developed jaundice, but all were within physiological limits for bilirubin. Sepsis was recorded in only one of the infants classified as a non-responder. Only one of our non-responding babies had prior antibodies, but in conjunction with severe intrauterine restriction and the receipt of multiple transfusions.

We found no differences in immune response in the infants with either IUGR, or in the different groups stratified according to percentile weight at 6 months, consistent with previous findings [15, 17]. However, at the time of vaccine initiation, GMCs were lower in those children who weighed less than 2 kg but this is logical given that they were smaller at birth.

Three doses of HB vaccines are generally recommended in preterm babies, although 4 doses have been suggested [10, 15, 17, 22, 29, 30], as well as an antibody test after the third to assess the suitability of a booster dose [19, 20, 22]. In a study of 29 preterm infants who were non-responders, 14 received additional doses of vaccine; 7 seroconverted after the first additional dose, 3 required a second additional dose and 1 required a third [16]. In another study although only three of the nine non-responding pre-term infants returned for a fourth vaccine dose, all subsequently seroconverted [26]. We decided to administer 4 doses, but according to the schedule recommended for full-term babies, and include a booster dose at 18 months with the hexavalent vaccine used for primary vaccination. All six infants that did not respond to the primary course also did not respond to a fourth dose; 1 infant responded to the fifth, 2 to the sixth, and 1 after seven doses. Only two infants remained non-responders. 

Few authors have studied the persistence of anti-HBs in premature infants; however, Kesler et al. [27] reported that similar declines in antibody titres are seen in pre-term and full-term babies after primary vaccination, although preterm infants generally have a lower titre. We found similar results prior to the booster dose and observed a good immune response with similar GMCs among preterm and full-term infants after the booster. 

Many long-term studies of infants indicate that immunisation against hepatitis B induces both antibody-producing-cells and memory cells, and the rapid development of anamnestic responses [31]. Booster doses of vaccine do not seem necessary to ensure long-term protection [32–34], although some factors might be important. Delaying vaccination until infants achieve a weight of 2000 g results in a significantly higher GMC at 3-3.5 year of age as compared to early vaccination in both pre- and full-term infants [35]. Most children vaccinated at birth retain immunological memory to hepatitis B vaccine for 15 years, but those who did not were more likely to have received HB IG immunoglobulin at birth, suggesting that passive antibody may interfere with the induction of immunological memory [36]. 

In summary, preterm babies born to HBsAg-negative mothers and vaccinated with a hexavalent DTPa-HBV-IPV/Hib vaccine at 2, 4, and 6 months responded well immunologically and similarly to children born at full term. The response in terms of seroprotection rates was not influenced either by gestational age or birth weight, and these findings strengthen the notion that the immunological response in premature babies depends on postnatal age and not on birth weight or gestational age. Six infants, all with a gestation age above 31 weeks, did not respond, but we did not find any associated risk factors, other than severe intrauterine malnutrition in two babies. After a further vaccination cycle, only 2 remained “true” non-responders. The vaccination schedule used here for preterm babies born to HBsAg-negative mothers offers a single schedule for all infants, with fewer injections and better acceptance.

## Figures and Tables

**Table 1:** Anti- HBs seroprotection rates and GMCs in preterm and full-term infants one month after the primary course and before and after the booster dose (ATP cohort for immunogenicity).

		Seroprotection			GMC	
	*N*	*n*	%	95% CI	mIU/ml	95% CI
Postprimary						
Preterm	91	85	93.4	86.2–97.5	634.1	433.8–927.0
Full-term	84	80	95.2	88.3–98.7	867.1	576.6–1303.9

Prebooster						
Preterm	84	63	75.0	64.4–83.8	56.8	39.6–81.4
Full-term	67	54	80.6	69.1–89.2	58.1	39.1–86.3

Postbooster						
Preterm	83	76	91.6	83.4–96.5	1771.0	1060.3–2958.1
Full-term	68	67	98.5	92.1–100.0	1965.0	1180.1–3272.0

**Table 2:** Anti-HBs seroprotection rates (SR) and geometric mean concentrations (GMC) by subgroup of preterm infants after primary course and booster dose (ATP cohort for immunogenicity).

		Seroprotection			GMC	
Gestational age (weeks)	*N*	*n*	%	95% CI	mIU/ml	95% CI
Primary course						
34–36	27	25	92.6	75.7–99.1	955.9	461.1–1981.8
31–33	24	20	83.3	62.6–95.3	418.0	153.7–1136.4
28–30	20	20	100	83.2–100	1044.5	633.9–1721
24–27	20	20	100	83.2–100	364.8	184.5–721.5

Booster dose						
34–36	24	22	91.7	73.0–99.0	2389.1	919.4–6207.9
31–33	23	18	78.3	56.3–92.5	1127.2	262.2–4845.2
28–30	18	18	100	81.5–100	3555.9	1881.1–6721.8
24–27	18	18	100	81.5–100	1054.0	508.6–2184.2

**Table 3:** Post-primary anti-HBs seroprotection rates and GMCs by birth weight (ATP cohort for immunogenicity).

		Seroprotection			GMC	
Birth weight (kg)	*N*	*n*	%	95% CI	mIU/ml	95% CI
≥2.0	17	14	82.4	156.6–96.2	493.9	147.2–1657.0
≥1.5<2.0	23	22	95.7	78.1– 99.9	933.8	418.8–2081.8
≥1.0<1.5	26	26	100	86.8–100	1294.5	882.5–1898.6
<1.0	25	23	92.0	74.0–99.0	250.7	122.0–515.1

**Table 4:** Post-primary anti-HBs seroprotection rates and GMCs by weight at vaccination and percentile weight at six months (ATP cohort for immunogenicity).

		Seroprotection			GMC	
	*N*	*n*	%	95% CI	mIU/ml	95% CI
Weight at first vaccination (kg)						
≥4	19	16	84.2	60.4–96.6	617.4	206.8–1843.8
≥3<4	26	25	96.2	80.4–99.9	984.0	479.0–2021.6
≥2<3	26	26	100	86.8–100	972.6	589.2–1605.3
<2	20	18	90.0	68.3–98.8	210.7	95.4–465.2

Percentile weight at six months						
>50	29	27	93.1	77.2–99.2	1140.8	577.6–2253.1
>25 ≤50	21	20	95.2	76.2–99.9	353.7	150.8–829.4
>10≤25	14	13	92.9	66.1–99.8	561.4	205.7–1532.4
≤10	27	25	92.6	75.7–99.1	566.2	280.0–1144.7

**Table 5:** Anti-HBs concentrations (mIU/ml) after all HBV doses in subjects who were non-responders to hepatitis B at the end of the primary and booster studies.

										Post Extra HBV			HBV
Gestational age (Weeks)	Birth weight (kg)	Percentile at birth	Weight at 1st vaccination (kg)	Percentile at 6 months	Weight at 6 months (kg)	Pre-primary	Post-primary	Pre-booster	Post-booster	Dose 1	Dose 2	Dose 3	Persistence at 4 years (mIU/ml)
33	2.1	75	4.4	95	8.3	<10	<10	<10	<10	690	3485	NG	176
35	2.0	25	4	25	6.7	<10	<10	<10	<10	<10	<10	32	128
32	0.6	<10	1.7	<5	4.4	<10	<10	<10	<10	<10	141	NG	64
31	0.8	<10	1.7	10	5.1	97	<10	<10	<10	<10	NA	NG	<10
33	2.0	50	4.3	>90	8.4	<10	<10	<10	<10	<10	<10	<10	<10
35	1.7	10	3.6	50	6.6	<10	<10	<10	<10	<10	1087	NG	NA

NA = Not available; NG = Not given.
